# The Reciprocal Relation Between Adolescents’ School Engagement and Alcohol Consumption, and the Role of Parental Support

**DOI:** 10.1007/s11121-015-0598-z

**Published:** 2015-09-04

**Authors:** Lukas Roebroek, Ina M. Koning

**Affiliations:** Child and Adolescent Studies, Utrecht University, P.O. Box 80.140, 3508 TC Utrecht, The Netherlands

**Keywords:** Alcohol use, Adolescents, Parental support, School engagement, Reciprocal effects

## Abstract

While school engagement and the use of alcohol are subject to change during the course of adolescence, studies have shown that being engaged in school equates with a later onset of alcohol consumption. Cross-sectional studies also indicate that alcohol use correlates to school engagement, but the reciprocal nature of these factors has never been investigated. This study examines the reciprocal relation between school engagement and alcohol consumption during adolescence. Further, the moderating effect of perceived parental support in this reciprocal relation between school engagement and alcohol consumption is tested. Data were obtained from Dutch high school students (*n* = 906, 52.5 % boys, mean age = 12.19 years) who annually completed a digital questionnaire over 4 years (age 12 to 15). A cross-lagged autoregressive model was applied in AMOS. Results showed that more school engagement at ages 12 and 14 predicted lower levels of alcohol use 1 year later. In addition, more alcohol consumption at ages 12 and 14 predicted lower levels of school engagement 1 year later. Higher school engagement at age 13 predicted less alcohol use at age 14, whereas no significant effect of alcohol use on school engagement was found at this age period. Furthermore, a reciprocal relation was found only for adolescents who perceived high parental support. The reciprocal nature of school engagement and alcohol consumption should be a consideration in future research and prevention program development.

It is essential for adolescents to be highly engaged in school, as low school engagement is an important risk factor for a variety of deviant behaviors (Li et al. 2011; Loukas et al. 2009; Simons-Morton et al. 1999). For instance, adolescents showing a low degree of school engagement are at greater risk of engaging in delinquent behavior (Liljeberg et al. 2011) and alcohol use in particular (Li et al. 2011). It is likely that drinking alcohol also influences school engagement, yet the nature of this reciprocal relation is unknown. It is imperative to understand this, as both factors are associated with different consequences. Excessive alcohol consumption at a young age, for instance, can cause physical and psychological problems, and can lead to a decrease in school performances (DeWit et al. 2000; Latvala et al. 2014). Adolescents who become disengaged from school are more likely to experience a wide variety of cognitive, behavioral, and emotional problems (Holt et al. 2008), such as school dropout, delinquency, and official offending (Henry et al. 2012). Therefore, it is crucial to gain more insight into the reciprocal relation between school engagement and alcohol consumption, and the effect parental support has upon it.

## School Engagement and Alcohol Use Over Time

School engagement of Dutch adolescents is subject to change during the course of their high school careers. Almost all (95 %) 12-year-old Dutch adolescents indicate that they either enjoy school a little or a lot. By the age of 16, this percentage has declined to almost 70 %. Further, judgements about performance in school change as students get older. Among 12-year-old Dutch adolescents, 72 % judge their own performance at school as either good or very good. By age 16, this percentage has dropped to 55 % (Van Dorsselaer et al. 2010). As adolescents mature, their positive school engagement tends to decline. Concurrently, involvement in risk behaviors, alcohol use in particular, increases during the course of adolescence (Hibell et al. 2012).

Alcohol consumption in Dutch adolescents rapidly increases from the moment they enter high school. About 35 % of 12-year-old students indicate that they have consumed alcohol. This percentage increases to about 90 % for 16-year-olds (Verdurmen et al. 2012). In addition, about 8 % of 12-year-old and 77 % of 16-year-olds indicate having consumed alcohol in the preceding month. When adolescents drink, they tend to drink a lot. For example, 3 % of all 12-year-olds indicate having engaged in binge drinking (≥5 glasses on a single occasion) in the previous month, which increases to 57 % of 16-year-olds. Excessive alcohol consumption at a young age can cause psychical and psychological problems and can lead to a decrease in school performances (DeWit et al. 2000). Furthermore, the earlier a student starts drinking, the greater the chances of alcohol abuse 10 years later (Behrendt et al. 2008). The percentage of Dutch students who drink rapidly increases the moment they enter high school, and by the end of high school, a majority of them drink frequently and substantially.

## The Relation Between School Engagement and Alcohol Consumption

Different studies have shown that a high degree of school engagement serves as a protective factor against problem behavior in general and alcohol consumption in specific (Chiarella 2003; Dever et al. 2012; Henry et al. 2009; Simons-Morton et al. 1999; Wu et al. 2007). Based on the social development model (Catalano and Hawkins 1996), adolescents tend to learn behavior patterns from their primary socialization sources like parents, peers, and school. A strong bond with pro-social sources, for instance, school, can serve as a protective factor against deviant behavior. On the other hand, a weak bond with pro-social sources and a strong bond with antisocial sources, for instance peers who engage in deviant behavior, is a risk factor for developing problem behavior. Therefore, it is expected and shown in previous research that adolescents with a strong school bond start drinking at a later age and have a smaller chance of becoming dependent upon alcohol (Maddox and Prinz 2003; Shears et al. 2006).

Relatively few studies have examined whether alcohol consumption has an effect on school engagement. Määttä et al. (2006) examined the effect of norm-breaking behavior, which also included alcohol consumption, on school engagement. They showed a negative effect of norm-breaking behavior on school engagement. Other research has shown negative correlations between alcohol consumption and school engagement (Tarter et al. 1996; Henry et al. 2009). Furthermore, there is substantial evidence that alcohol use predicts and is predicted by lower school achievement across adolescence (e.g., Crosnoe 2006; Hayatbakhsh et al. 2011; Latvala et al. 2014). Academic achievement and school engagement are two related, yet different factors (Wong and Holcombe 2010) relevant for understanding academic success; school engagement tends to precede academic performance (Wong and Holcombe 2010). So far, previous studies have not been able to make a clear statement about the reciprocal nature of the relation between alcohol consumption and school engagement. There is a solid theoretical framework to substantiate the notion that a high degree of school engagement can serve as a protective factor against alcohol consumption for adolescents. However, there is a lack of evidence to verify a negative effect of alcohol consumption on school engagement. Most research on this topic has been done using cross-sectional data, which makes it impossible to draw conclusions about the exact relation between school engagement and alcohol consumption. By using longitudinal data, this study offers new insights into the entangled relation between school engagement and alcohol use.

## The Role of Parental Support on School Engagement and Alcohol Consumption

Parents play an important role in their children’s school engagement and alcohol consumption, and parents’ influence can be measured in both areas (Chaplin et al. 2012). Adolescents that receive more warmth and support from their parents drink significantly less and hold a more positive attitude towards school in comparison to adolescents who feel less warmth and support from their parents (Bogenschneider et al. 1998; Chaplin et al. 2012; DeSantis King et al. 2006; Wills et al. 2004). A supportive relation with parents can serve as a protective factor against forming deviant peer relations, which in turn, can lead to a reduction in alcohol use (Wu et al. 2007). Parents can also motivate their children to do well in school. Wu et al. (2007) suggested that this could be a possible explanation why adolescents who have a close bond with their parents show a higher degree of school engagement as compared to adolescents who have a weaker bond with their parents. Though parental support relates to higher levels of school engagement and lower levels of drinking, it is unknown whether the relation between the latter factors differs according to the level of parental support. Previous research has revealed the moderating effect of parental support on the relation between adolescents’ negative affect and subsequent alcohol use (Reimuller et al. 2011). As parents become effective targets in preventive intervention programs, it is important to have insight into the role of parents in the relation between school engagement and alcohol use. This is the first study to examine whether parental support moderates the relation between school engagement and alcohol consumption.

## Current Study

This study aims to examine (1) the reciprocal nature of the relation between school engagement and alcohol consumption and (2) whether this relation is moderated by the degree of parental support. We hypothesize that a higher degree of school engagement leads to less alcohol consumption and more alcohol consumption leads to a lower degree of school engagement. In addition, we expect that the relation between alcohol consumption and school engagement is stronger among adolescents with less supportive parents. The investigation of the reciprocal relation between alcohol use, school engagement, and the role parental support plays may provide an important new argument for implementing more stringent alcohol prevention programs for adolescents entering high school with a potentially import role for parents.

## Method

### Procedure and Participants

In April 2006, out of a list of all Dutch public secondary schools, 80 schools were randomly selected and invited to participate in the intervention study: “Preventing heavy alcohol use in adolescents” (PAS: Koning et al. 2009). Schools were allowed to participate if the following inclusion criteria were met: (i) at least 100 first-year students, (ii) less than 25 % students from migrant populations, and (iii) no special education was offered. Nineteen schools from different regions and neighborhoods in the Netherlands participated in this study. From these schools, all first-year students (*n* = 3490) were asked to participate. Schools were randomly assigned to one of four groups: three experimental and one control. Students in the experimental groups all received some form of intervention aimed at reducing alcohol use. Therefore, the data from the control group were of particular interest to this study; four schools comprising 47 classes with a total of 935 students participated (average school size = 233 students). Participating high schools represent all different educational levels in the Netherlands, from prevocational education to pre-university secondary education.

A trained research assistant obtained data from the students. The data were collected in classrooms by means of an online questionnaire that was made available on a secure website. The students who participated in this study filled in these questionnaires on a yearly basis from 2006 to 2009 (T1 to T4). Parents received a letter of consent and were given the opportunity to refuse their child’s participation.

Because of initial non-response among adolescents at the time 1 (T1) (*n* = 29), 906 adolescents were eligible for analysis. Non-response at each wave occurred due to student absence on the day of the assessment or because students moved away. Of the 906 students that did participate in the first wave, 476 (52.5 %) were boys, (60.2 %) were in lower secondary education. Almost one fifth of the adolescents (18 %) reported to live in a single-parent family, which is in accordance with the national percentage of 19 % (CBS 2011). The average age at the first wave was 12.19 years old (SD = 0.51).

### Loss to Follow-Up

Adolescents who did not participate at T2 (42 = 4.6 %), T3 (104 = 11.5 %), or T4 (123 = 13.6 %) differed as compared to completers at baseline, with higher amounts of alcohol consumption for those lost to follow-up at T3 (*t* = 2.83, *p* = 0.006) and T4 (*t* = 2.29, *p* = 0.024), with a lower degree of school engagement for those lost to follow-up at T2 (*t* = −2.12, *p* = 0.035), a lower level of education for those lost to follow-up at T3 (*X*^*2*^ = 18.24(1), *p* < 0.001) and T4 (*X*^*2*^ = 16.67 (1), *p* < 0.001) and with a higher average age for those lost to follow-up at T3 (*t* = 2.26, *p* = 0.025) and T4 (*t* = 2.52, *p* = 0.013). No gender differences were found for those not participating at T2 (*X*^*2*^ = 0.09 (1), *p* = 0.77); T3 (*X*^*2*^ = 0.12 (1), *p* = 0.73); and T4 (*X*^*2*^ = 2.19 (1), *p* = 0.139) as compared to the completers.

### Outcome Measures

#### Weekly alcohol consumption

Students were asked at four time points how many days during the week (i) and on weekends (ii) did they usually drink alcohol and, how many glasses did they usually drinks in a typical week (iii) and weekend day (iv). The questions are part of the Quantity-Frequency Index, which is used to determine weekly alcohol consumption (Straus and Bacon 1953). The scores of items i and iii are multiplied; the scores of items ii and iv are also multiplied. These two scores are then summed to represent the average weekly alcohol consumption. Some students reported unrealistically high average weekly alcohol consumption scores (e.g., 104 glasses per week). These scores were all reduced to the mean plus three standard deviations (T1 = 12, T2 = 27, T3 = 40, T4 = 45).

#### School engagement

The degree of school engagement was also assessed at each wave. The following five statements were presented: (i) do you enjoy school, (ii) do you try your best at school, (iii) do you feel like you are forced to be in school, (iv) are you satisfied with your homework, and (v) do you find schoolwork easy. These statements were to be answered on a 5-point scale ranging from *almost never*, *mostly not*, *sometimes*, *mostly yes*, and (*almost*) *always*. Items i to v were calculated into a single average score for every student, on each consecutive time point, representing the average degree of school engagement. Scores ranged from 1 to 5, with a score of 1 indicating the lowest degree of school engagement and a score of 5 indicating the highest degree of school engagement. To increase the internal consistency of this scale, item iii was removed from the analysis. The Cronbach’s alpha ranged from 0.68 to 0.72.

#### Parental Support

In order to assess the degree of parental support students perceive, the “easy to talk to” scale was used (Van Dorsselaer et al. 2010). Students were asked at T1 how easy is it for them to talk about things that worry them with (i) their father, (ii) step-father (or mother’s new partner), (iii) mother, and (iv) step-mother (or father’s new partner). Answers ranged on a 5-point scale with the categories being, *very easy*, *easy*, *difficult*, *very difficult*, or *don*’*t have*/*never see*. This last category, don’t have/never see, is scored as a missing value. For every student, a mean score was calculated (range 1 to 4); a higher score indicated more parental support. When a score of both father and mother was present, the mean was calculated with the use of these two scores. When a score for either father, mother, or father and mother was missing, the score of the step-father and or step-mother was used when present to calculate the mean. The degree of parental support is dichotomized by means of a median-split (median = 3; ≤3 = 39.3 %, ≥3 = 61.7 %) to allow for multi-group analysis. The *ρ* for these two items is 0.61 (*p* < 0.001).

### Analysis

**Fig. 1:**
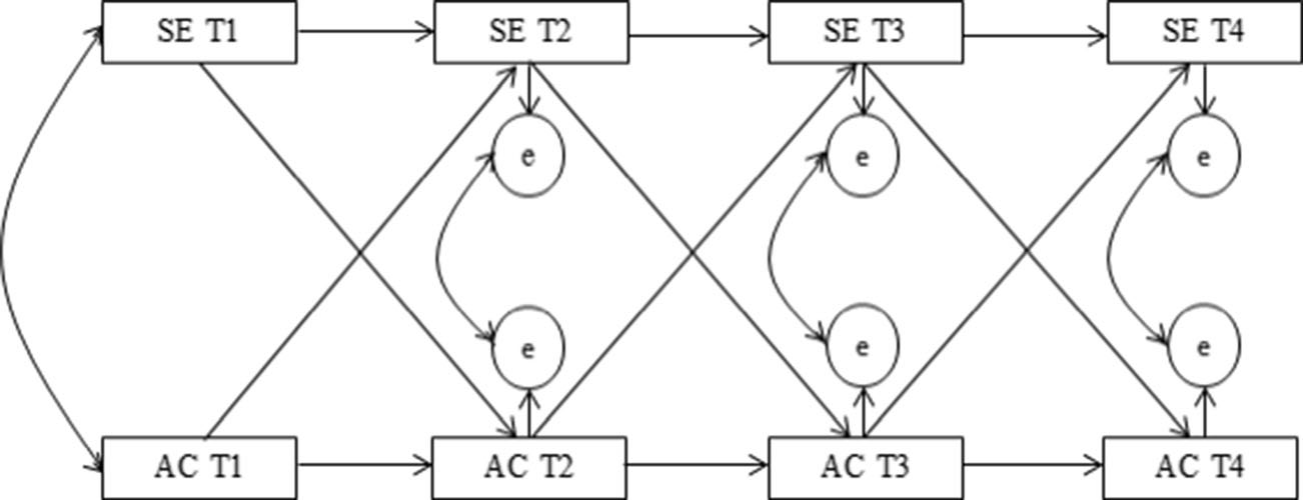
Theoretical Model. *SE* school engagement, *AC* alcohol consumption

In order to test the three different hypotheses, structural equation modeling was conducted using AMOS (Fig. 1). This model consisted of the following characteristics: (i) the value for time point T can be explained by the value of time point T1, (ii) the correlations between the error terms that are generated by the dependent and independent variables are controlled, and (iii) the time-dependent pathways for both school engagement and alcohol consumption are added in order to control for the stability of each variable. AMOS handles missing values by making use of full information maximum likelihood estimates. This method makes an estimate for each missing value that is used in the analysis (Enders and Bandalos 2001).

Frequency tables are used to present the mean and standard deviations of the different variables of interest. The first analysis serves to test the first and second hypotheses. A cross-lagged model was used to test the reciprocal relation between school engagement and alcohol consumption.

In order to test the third hypothesis, moderation by parental support, previous analyses were carried out for both the low- and high-support group.

## Results

### Descriptive Data

The descriptive statistics of both school engagement and alcohol consumption are presented in Table 1. The average school engagement decreased over time. The average alcohol consumption strongly increased as adolescents get older.

**Table 1:** Means and standard deviations of school engagement and alcohol consumption across all four time points

	M (SD)	M (SD)
	School engagement	Alcohol consumption
T1	3.90 (0.65)	0.55 (1.80)
T2	3.81 (0.71)	1.43 (4.56)
T3	3.66 (0.71)	2.64 (7.29)
T4	3.64 (0.70)	5.44 (9.94)

Table 2 shows the significant correlations between school engagement and alcohol consumption across all four time points. School engagement at T2 and T3 and alcohol consumption at T1 and T3 showed the strongest correlations. School engagement was significantly negatively correlated to alcohol consumption across each of the time points, ranging from *r* = −0.21 to *r* = −0.34.

**Table 2:** Correlations between school engagement (SE) and alcohol consumption (AC) across the four time points

	SE T1	SE T2	SE T3	SE T4	AC T1	AC T2	AC T3
SE T1	X						
SE T2	0.36**	X					
SE T3	0.32**	0.53**	X				
SE T4	0.24**	0.35**	0.44**	X			
AC T1	−0.23**	−0.21**	−0.10**	−0.15**	X		
AC T2	−0.17**	−0.34**	−0.18**	−0.14**	0.56**	X	
AC T3	−0.16**	−0.25**	−0.21**	−0.16**	0.28**	0.42**	X
AC T4	−0.09**	−0.24**	−0.21**	−0.21**	0.27**	0.40**	0.49**

**p ≤ 0.01

### The Reciprocal Relation Between School Engagement and Alcohol Consumption

**Fig. 2:**
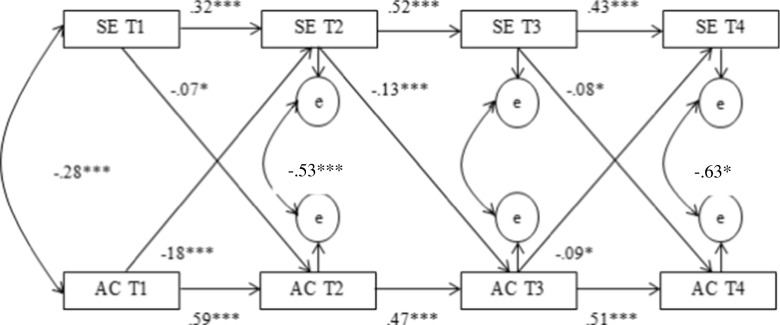
Cross-lagged, autoregressive model for the reciprocal influences between school engagement and alcohol consumption. *p ≤ 0.05, ***p ≤ 0.001. Only significant paths are depicted

In order to demonstrate the reciprocal relation between school engagement and alcohol consumption among adolescents, a cross-lagged autoregressive analysis was used. Figure 2 shows the results of this analysis along with the standardized regression coefficients belonging to the different paths. This model fits the data reasonably well (*χ*^2^ = 74.6 (12), comparative fit index (CFI) = 0.95, root mean square error of approximation (RMSEA) = 0.08).

School engagement and alcohol consumption influenced each other reciprocally in this model. School engagement had a negative effect on alcohol consumption across all three time points (*r* = −0.07, *p* = 0.019, *r* = −0.13, *p* < 0.001 and *r* = −0.08, *p* = 0.03). Alcohol consumption had a negative effect on school engagement across two time points, which continued into the next (*r* = −0.18, *p* < 0.001 and *r* = −0.09, *p* = 0.014) except the period from T2 on T3.

### Moderation of Parental Support

Table 3 presents the average school engagement and the average alcohol consumption for both adolescents who perceive a high degree and a low degree of parental support. School engagement among adolescents in the high-support group was significantly higher at T1 (*t* = −4.52, *p* < 0.001); T2 (*t* = −4.64, *p* < 0.001); T3 (*t* = −2.93, *p* = 0.03); and T4 (*t* = −2.43, *p* = 0.015) compared to adolescents in the low-support group. Alcohol consumption of adolescents in the high-support group was significantly lower at T1 (*t* = 2.90, *p* = 0.004) and T2 (*t* = 2.55, *p* = 0.011) compared to adolescents in the low-support group.

**Table 3:** Means and standard deviations of school engagement and alcohol consumption for both low parental support group and the high parental support group across all four time points

	M (SD)	M (SD)
	Low support	High support
SE T1	3.79 (0.62)	3.98^a^ (0.63)
SE T2	3.69 (0.72)	3.92^a^ (0.68)
SE T3	3.56 (0.72)	3.72^a^ (0.72)
SE T4	3.56 (0.65)	3.69^a^ (0.73)
AC T1	0.80 (2.34)	0.37^a^ (1.33)
AC T2	1.96 (5.59)	1.00^a^ (3.46)
AC T3	3.24 (7.84)	2.30 (7.02)
AC T4	6.12 (10.35)	4.96 (9.79)

^a^Significantly different from the low-support group at *p* < 0.05

**Fig. 3:**
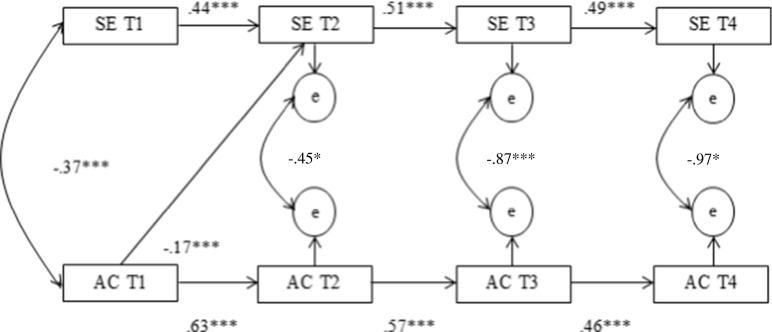
Cross-lagged, autoregressive model for the reciprocal influences between school engagement and alcohol consumption for the low parental support group. *p ≤ 0.05, ***p ≤ 0.001. Only significant paths are depicted

**Fig. 4:**
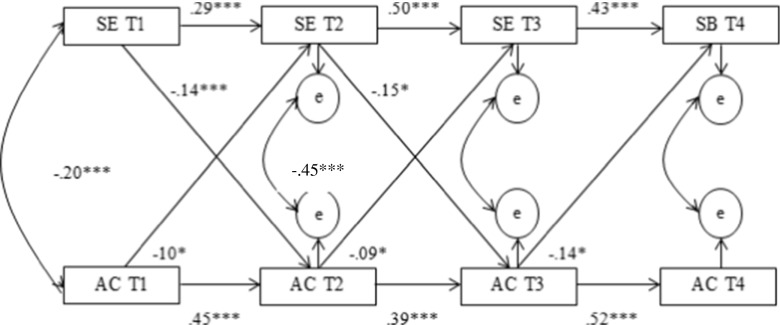
Cross-lagged, autoregressive model for the reciprocal influences between school engagement and alcohol consumption for the high parental support group. *p ≤ 0.05, ***p ≤ 0.001. Only significant paths are depicted

In order to test whether the perceived degree of parental support moderates the relation between school engagement and alcohol consumption, the earlier introduced cross-lagged model was tested for the low-support group (Fig. 3) and high-support group (Fig. 4). Both models fit the data well (Fig. 3; *χ*^2^ = 47.6 (12), CFI = 0.94, RMSEA = 0.09 and Fig. 4; *χ*^2^ = 45.2 (12), CFI = 0.95, RMSEA = 0.07).

In the model, for the low-support group, alcohol consumption at T1 had a negative effect on school engagement on T2 (*r* = −0.17, *p* < 0.001). No other significant effects of alcohol use and school engagement were found.

The model for the high-support group showed that alcohol consumption had a negative effect on school engagement across all three points in time (*r* = −0.10, *p* = 0.023; *r* = −0.09, *p* = 0.04; and *r* = −0.14, *p* = 0.001). School engagement at T1 and T2 had a negative effect alcohol consumption 1 year later (*r* = −0.14, *p* < 0.001 and *r* = −0.15, *p* = 0.001).

## Discussion

This is the first study to examine the reciprocal relation between adolescents’ school engagement and alcohol consumption, and the moderating effect of parental support. The results of this study support our hypotheses for the most part. School engagement has a negative effect on alcohol consumption over time. Alcohol consumption has a negative effect on school engagement at two of the three points in time. This study shows that school engagement and alcohol consumption influences one another reciprocally. Yet, this reciprocal relation is only true for adolescents who experience a high degree of parental support.

The reciprocal relation between school engagement and alcohol consumption adds an important new insight to previous research. In line with previous research (Chiarella 2003; Dever et al. 2012; Henry et al. 2009; Simons-Morton et al. 1999; Wu et al. 2007), the current study revealed that lower school engagement predicts more alcohol use. Yet, this contradicts the findings of Latvala et al. (2014) with respect to academic achievement and alcohol use; in that study, lower academic achievement in adolescence did not influence future drinking. This underlines the importance for continued research to investigate different school factors in relation to alcohol use during adolescence, such as academic achievement and school bonding. Furthermore, the results of this current study are in line with the study of Määttä et al. (2006) on the impact of norm-breaking on school engagement, by showing a negative effect of alcohol consumption on school engagement. These results add an important new insight to previous research by uncovering the reciprocal nature of the relation between school engagement and alcohol consumption. Further studies are needed on the reciprocal nature of the relations between these two variables.

The reciprocal relation between school engagement and alcohol consumption only applies to adolescents who perceive a high degree of parental support. School engagement and alcohol consumption have little effect on one another among adolescents who perceive a low degree of parental support. We postulate two explanations for this finding. One possible explanation could be the so-called ceiling effect. When score distributions tend to be skewed, regression could lead to inaccurate predictions of a certain variable (Kennedy 1998). As shown in Table 3, the low-support group has a lower average school engagement and higher average alcohol consumption at all four time points when compared with the high-support group. The effect of school engagement on alcohol consumption and vice versa could therefore be smaller because there is little room for change over time. A second explanation may be found in the difference of the degree of deviant peer relations between adolescents who perceive a high and low degree of parental support. Wu et al. (2007) showed that a good relation with parents can serve as a protective factor against the formation of deviant peer relations among adolescents, which in turn, is associated with less alcohol consumption. Marschal and Chassin (2000) also demonstrated that parental support plays a pivotal role in the socialization processes among adolescents. Parental support strengthens the positive intrapersonal skills among adolescents, which are vital in order to withstand the pressures of peers who encourage the use of alcohol. Adolescents who perceive a high degree of parental support might be more skilled to withstand the influence of high risk-taking peers in comparison to adolescents who perceive a low degree of parental support. It is possible that low parental support correlates with intrapersonal skills to cope with peer pressure. Without the necessary skills, adolescents perceiving low parental support may be more vulnerable to negative peer influences. More research is needed to examine the role of (high risk taking) peer relations in the relation between school engagement and alcohol consumption.

### Strengths and Limitations

The generalizability of these findings may be limited by a number of issues. First, school engagement and alcohol consumption have been measured using self-report questionnaires, which could lead to socially desirable answers. Previous research has shown that using self-report questionnaires is a reasonably reliable method for measuring alcohol consumption (Koning et al. 2010; Wagenaar et al. 1993). In order to guarantee a reliable measure of school engagement, future research might consider using teacher assessments in combination with self-report questionnaire. Second, this study does not provide a full explanation of why the degree of parental support moderates the reciprocal relation between school engagement and alcohol consumption because possible covariates were not controlled for. Future research could, for instance, control for the socio-economic status of students’ parents. Hartas (2011) showed that parental support serves as a pathway through which socio-economic factors influence children’s competencies. Parental support might thus be part of a latent variable like socio-economic status of the parents. Furthermore, more research is suggested on the mediating and moderating role that parents may play in the alcohol use–school engagement relation to improve our understanding of the mechanisms involved and how these may change based on risk status. Third, in the current cross-lagged model, we did not account for the long-term mean increase in alcohol use and decrease in school engagement. A bivariate latent growth model could be used in future research to capture the trajectories of alcohol use and school engagement. Last, the aim of the study was to examine differential effects for high versus low support from parents, whereas it would be interesting to examine tertiles (low, average, high) or the linear scale of parental drinking in future studies.

Despite these limitations, this study examines the reciprocal nature of the relation between school engagement and alcohol consumption by making use of longitudinal data and a large sample of adolescents. In particular, the effects of alcohol consumption on school engagement are revealed, which have been addressed in previous research. Furthermore, this study is the first to show the moderating effects of parental support on the relation between school engagement and alcohol consumption. In sum, this study is first to show that alcohol use both predicts and is predicted by lower school engagement, yet only for adolescents who perceive a high degree of parental support.

### Implications

The current study has important implications for future research, practice, and policy. As indicated above, it is important for future research to investigate different school factors in relation to adolescents’ alcohol use. The current study examined the relation between school engagement and alcohol use. Yet, other factors such as academic achievement and school bonding, both predictive of alcohol use (e.g., Bryant et al. 2003), should be taken into account, as these factors may be differently related to drinking during adolescence. Furthermore, this study demonstrated the importance of investigating the two-way relation between alcohol use and its risk factors.

Early alcohol use is not only predicted by lower school engagement, but also predicts subsequent lower school engagement. This reciprocal relation has far-reaching consequences for students, as school engagement is predictive of academic success and subsequent career opportunities later in life (Upadyaya and Salmela-Aro 2013). Moreover, this underlines the importance to postpone the onset of drinking among young adolescents who enter high school, by implementing an effective alcohol prevention program that targets parents as well as adolescents, such as the Prevention of Alcohol use in Students program (PAS; Koning et al. 2009). PAS effectively postponed the onset of (heavy) weekly drinking among early adolescents up to 52 months after baseline. This is significant not only because an earlier onset of drinking increases the likelihood of alcohol abuse 10 years later (Behrendt et al. 2008), but also because early drinking leads to a decrease in school engagement, which in turn leads to even more alcohol consumption. Further, the PAS program can be improved by making an effort to stimulate adolescents’ school engagement from the moment they enter high school. Increasing school engagement at the same time serves as a protective factor against early drinking. Finally, the role of parental support has to be taken into account. Adolescents who perceive a low degree of parental support might have more trouble resisting negative influences from deviant peers because they lack the required intrapersonal skills, which are strengthened by parental support. Therefore, prevention programs should also pay extra attention to the development of intrapersonal skills among young adolescents that are essential to effectively deal with peer pressure.
